# Is There Evidence for the Development of Sex-Specific Guidelines for Ultramarathon Coaches and Athletes? A Systematic Review

**DOI:** 10.1186/s40798-022-00533-9

**Published:** 2023-01-25

**Authors:** Claudia P. M. G. Kelly

**Affiliations:** College of Medicine and Health, The University of Exeter, St Luke’s Campus, Heavitree Road, Exeter, EX1 2LU UK

**Keywords:** Ultramarathon, Female athletes, Physiological sex differences, Running performance

## Abstract

**Background:**

There is evidence of sex differences in the physiology of endurance exercise, yet most of the advice and guidelines on training, racing, nutrition, and recovery for ultramarathons are based on research that has largely excluded female athletes. The objective was therefore to review the current knowledge of sex differences in ultramarathon runners and determine if sufficient evidence exists for providing separate guidelines for males and females.

**Methods:**

This systematic review was carried out in accordance with the Preferred Reporting Items for Systematic Reviews and Meta-Analyses (PRISMA) guidelines. Three databases were searched for studies investigating differences in elite and recreational male and female ultramarathon runners. Studies were included if they compared males and females and looked at outcomes relating to the performance or health of ultramarathon runners. The quality of the included studies was determined using the Grading of Recommendations Assessment Development and Evaluation (GRADE) approach.

**Results:**

The search strategy identified 45 studies that met the inclusion criteria. Most studies were observational in design, with only three papers based on randomised controlled trials. The overall quality of the evidence was low. Sex differences in the predictors of ultramarathon performance; physiological responses to training, racing, and recovery; chronic and acute health issues; and pacing strategies were found. There were areas with contradictory findings, and very few studies examined specific interventions.

**Conclusion:**

The results from this review suggest that the development of sex-specific guidelines for ultramarathon coaches and athletes could have a significant effect on the performance and health of female runners. At present, there is insufficient high-quality evidence on which to formulate these guidelines, and further research is required.

## Key Points

1. Sex differences in ultramarathon runners have been demonstrated in a number of areas including the predictors of performance, fatigue resistance, susceptibility to injury and illness, and oxidative stress. This indicates that sex-specific approaches to race preparation and recovery could benefit female ultramarathon runners.
2. The evidence base comprises mostly of observational studies, and the level of evidence is generally low. The lack of experimental studies, relatively low number of female subjects, and the heterogeneity inherent in ultramarathon race formats makes it difficult to draw conclusions. Thus, it is not currently possible to formulate evidence-based, sex-specific guidelines for ultramarathon runners. Further interventional studies are required to examine sex differences in the physiological responses to different training, nutrition, and recovery modalities. Furthermore, the effects of these interventions during different phases of the menstrual cycle should be elucidated. Future research should also compare males with both pre- and post-menopausal females, as it is likely that female sex hormones significantly influence many study outcomes. Such research could guide the development of guidelines that could optimise female athlete performance and health.

## Introduction

Ultramarathons are running races involving distances greater than a marathon (> 42.195 km). Race formats vary widely, and races may take place on a variety of terrains including roads, trails, running tracks, and mountains. The popularity of these events has increased exponentially in the past three decades, including a significant increase in female participation [1]. Many female athletes have excelled at the sport, with some winning races outright, and this has sparked considerable interest and debate regarding the possibility that females hold a physiological advantage in ultraendurance sports [2, 3].

Ultramarathon performance is influenced by a complex interaction between various physiological and psychological factors [4, 17]. These include oxidative capacity, running economy, substrate utilisation, fatigue resistance, gastrointestinal function, motivational factors, pacing, nutrition, pain perception, age, and experience, [4–9, 17]. In recent decades, increasing evidence of sex-dependent differences in many of these variables has emerged [2, 10–14, 17]. Furthermore, the influence that these variables have on performance is likely to be mediated by sex, as different predictors of ultramarathon performance have been found in males and females [15, 16].

Despite this increasing recognition of the sex differences in ultramarathon running, the majority of research into health and performance interventions in endurance athletes has been carried out in males [2, 10, 13]. A recent study of papers published in the journal Applied Physiology, Nutrition, and Metabolism (APNM) between 1993 and 2021 found that, of 2547 papers, only 11% identified females as the primary intervention group [11]. Furthermore, only 35% of papers included both males and females, and only 10% of papers included sex in bivariate analyses [11]. The reasons behind this are likely complex and varied, including traditional attitudes and societal norms regarding females’ place in sport, and the added work and cost involved in controlling for variations in sex hormones associated with the menstrual cycle [18]. Additionally, it has been posited that sex differences in psychology result in males being more willing to participate in exercise and sports science research, contributing to their greater representation in the literature [19]. However, it is not known to what degree this increased willingness reflects biological or social influences on psychology. Regardless of the reason, the outcome is the same: female athletes have been following advice and guidelines regarding training, nutrition, and recovery that are based on studies that have largely excluded their sex [18].

Given the aforementioned sex differences, it is possible that the performance of female ultrarunners has not been enhanced by advances in scientific knowledge to the same degree as males. Moreover, there may be undesirable health consequences of following this overgeneralised approach. For example, research in male athletes has shown potential benefits of endurance training in a fasted or low carbohydrate availability state on measures such as mitochondrial density and lipid oxidation rates [20]. This contributed to the popularity of becoming “fat-adapted” among ultramarathon runners, an approach to nutrition and training which aims to improve the body’s ability to oxidise fat, sparing muscle glycogen and delaying fatigue. However, subsequent research has shown that within-day energy deficits, such as those involved in fasted training, are associated with clinical markers of metabolic and menstrual disturbances in female endurance athletes [21].

There is currently a paucity of sex-specific guidelines for ultramarathon runners and their coaches. This could be disadvantaging female athletes from a performance perspective, and undermining their health. Therefore, the purpose of this review is to summarise the current knowledge of sex differences in ultramarathon runners and determine if sufficient evidence exists for providing separate guidelines for males and females.

### A Note on Sex and Gender

Within this text, the terms “male”, “female”, “man/males”, and “woman/females” will be used to refer to sex, not gender. It is important to distinguish the differences between the terms “sex” and “gender”, which have historically been conflated within the literature [11]. Sex refers to the biological attributes of an individual, the physical and physiological features determined by genetics and hormone function, which are generally classified as female or male [22]. In contrast, gender is a social construct and relates to the roles, behaviours, and identities that an individual fulfils in society [22]. Gender exists on a spectrum and does not necessarily concur with the biological sex of an individual. As mentioned, the terms have been inappropriately used interchangeably when reporting scientific findings regarding biological sex differences. Thus, to prevent the exclusion of potentially relevant studies, both terms were used in the search strategy outlined in the methods.

## Methods

A systematic review was carried out in accordance with the Preferred Reporting Items for Systematic Reviews (PRISMA) guidelines [23]. The methods were specified in advance and documented in a detailed protocol.

### Study Inclusion and Exclusion Criteria

Inclusion criteria:

- Participants: Male and female ultrarunners (defined as individuals who have completed a race > 42.2 km) aged 18 years and above, who compete or participate in single-day or multi-day events.
- Interventions: Training, nutrition, racing, or recovery strategies. Observational studies with no intervention were also included.
- Comparisons: Males versus females.
- Outcomes: Injury and/or illness rates, performance (time to complete a given distance, ability to complete a race, race placing, average running speed, pacing across a given distance), and physiological parameters.
- Publication characteristics: Primary research published in the English language, or with an English translation.

Exclusion criteria:

- Races which also include cycling, swimming, or any other sport.
- Races ≤ 42.2 km.

### Search Strategy for Identification of Studies

The following electronic databases were searched on 27 September 2021: PubMed, SPORTDiscus, and Scopus. In addition to this, the citations list for each study and any reviews identified from the database searches were reviewed to identify other relevant papers. The search terms and combinations used were as follows:

(“ultramarathon*" OR “ultra-running” OR “ultra-marathon*” OR "ultra-endurance running" OR “ultrarunning" OR “ultrarunner*" OR “ultra-runner*" OR "ultrarun*" OR “ultra trail*”).

AND (“sex” OR “gender” OR "sex factor*" OR "sex specific" OR “sex-specific” OR "sex difference*" OR "gender difference*" OR "sex-based difference*")

AND (“physiology” OR “metabolism” OR “nutrition” OR “diet*” OR “fuel*” OR “train*” OR "endurance train*" OR “recover*” OR "race strategy" OR “racing” OR “performance” OR “illness” OR “injur*” OR “health”)

Screening: Studies identified in the search were screened according to a two-step process. First the titles and abstracts were reviewed and articles that either clearly did not meet the inclusion criteria or met any of the exclusion criteria were removed. Secondly, the full texts of all remaining studies were reviewed with further exclusions made accordingly.

### Quality Assessment

Study quality was assessed in accordance with the Grading of Recommendations Assessment Development and Evaluation (GRADE) framework [24].

## Results

### Study Selection

**Fig. 1:**
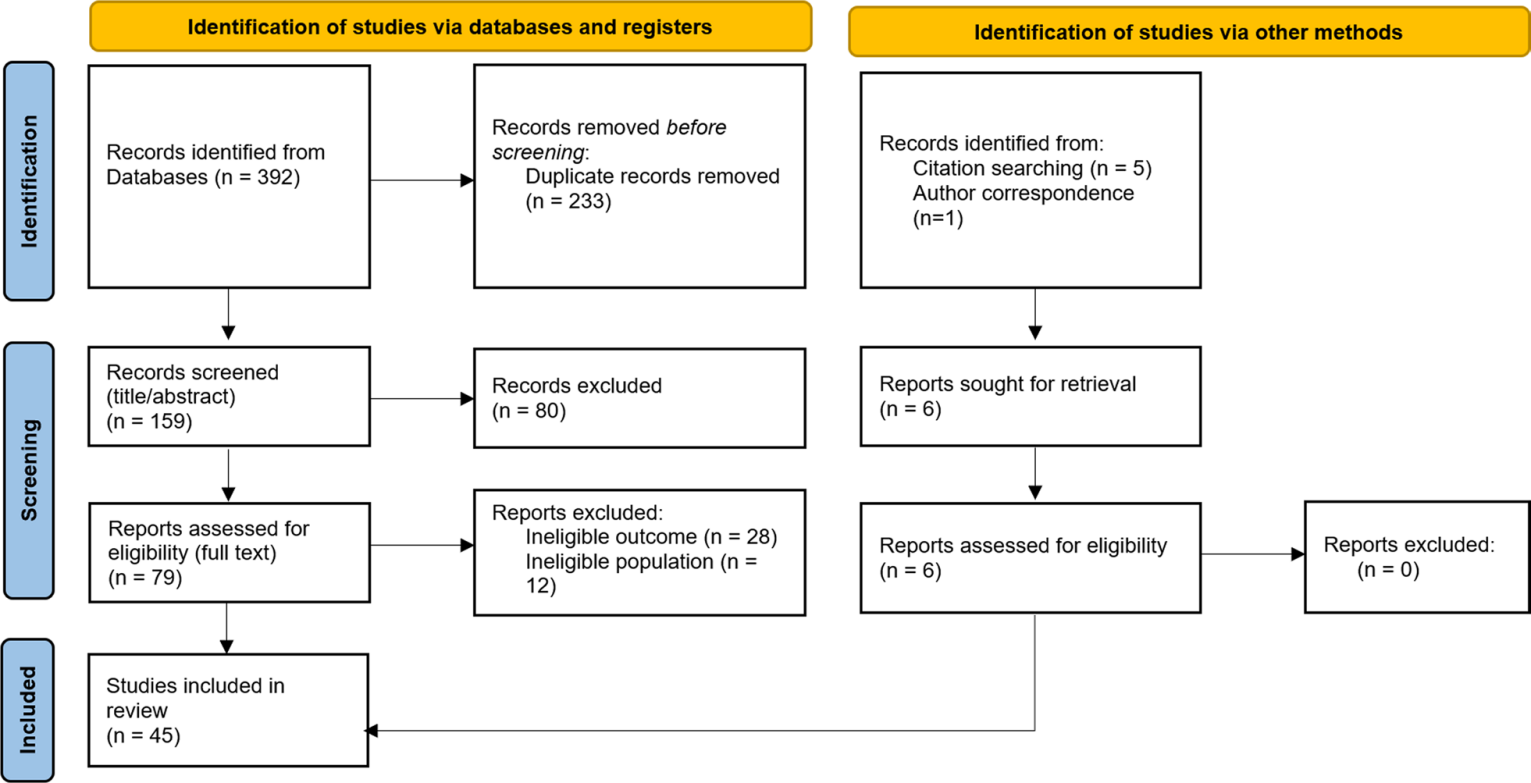
Summary of search strategy

The searches produced a total of 392 records. After duplicates were removed, there were 159 records remaining. The abstracts of these records were then screened, and 80 records were excluded. The full texts of the remaining 79 records were assessed for eligibility, with 39 studies identified as eligible. Six studies were identified from additional sources, including five from the citations lists of other eligible studies, and one via correspondence with an author. Therefore, a total of 45 studies were included in this review. A diagram summarising this process can be seen in Fig. 1.

### Study Characteristics

The study population, methods, and relevant findings from each of the included studies are summarised in Tables 1–8. The studies have been grouped into eight categories depending on their primary outcome measures. The number of studies in each of the eight categories are as follows: (I) predictors of performance—6 studies, (II) immune and oxidative stress—6 studies, (III) neuromuscular fatigue and cognition—3 studies, (IV) cardiopulmonary physiology—5 studies, (V) other physiology—3 studies, (VI) chronic health and lifestyle factors—4 studies, (VII) training or race-related illness and injury—12 studies, and (VIII) pacing strategy—6 studies.

Studies included in this review were methodologically varied; however, the vast majority were observational, with only three randomised controlled trials (RCTs) being included. This reflects the body of the literature on ultramarathons, where RCTs are rare [25]. Of the observational studies, there were 29 cohort studies, 10 cross-sectional studies, and 3 retrospective data analyses.

The quality of included studies reflects the preponderance of observational designs and small sample sizes. Despite this review selecting studies that compared males and females, females only comprised 35.1%, 27.2%, 50.7%, 36%, 21.3%, 27%, and 12.1% of subjects in the studies in categories I, II, III, IV, V, VI, and VIII, respectively. Due to the lack of reporting of the sex distribution of subjects for several of the studies in category VII, it was not possible to determine the overall proportion of females in this group. Hence, the evidence from 42 studies was graded as low quality, and only the three included RCTs were graded as providing high-quality evidence.

### Main Findings

#### Predictors of Performance

Six studies investigated sex differences in the physiological predictors of ultramarathon performance in 228 subjects, 80 of whom were female (Table 1) [15, 16, 26–29]. These studies looked at a wide range of parameters, including limb strength, cardiopulmonary function, body composition, and substrate utilisation. Five of the six studies found sex differences in the predictors of performance. Martinez-Navarro et al. found that mean inspiratory pressure was correlated with performance in males only (*r* = − 0.576, *p* = 0.010) [28]. In females, the Leg Qindex was the only variable that correlated with performance (*r* = − 0.607, *p* = 0.028) [28]. In another paper, Martinez-Navarro et al. reported that Vpeak, or the top speed measured during treadmill cardiopulmonary exercise testing (CPET), and maximal fat oxidation rate (MFO) together explained 66% of the variation in 107 km race time in males [16]. Conversely, in females, the V̇O_2_max alone accounted for 69% of race time variation [16]. When athletes involved in a shorter race were studied, Vpeak and V̇O_2_max correlated with performance in both males and females [29]. Another study by O’Loughlin et al. reported that age, BMI, average training speed, and years of running experience were related to ultramarathon performance in males, but not females, and females’ performance was predicted by training volume and personal best times in shorter races [15]. Of the four studies examining anthropomorphic measures, one found an association between body fat percentage and running speed in males only, and two found a similarly male-only relationship between performance and BMI, while the final study found no link in either sex [15, 26, 27, 29].

**Table 1:** Predictors of performance

Study	Participants	Measures	Design	Evidence quality	Major findings
Martinez-Navarro et al.[28]	32 athletes who completed a 107 km mountain ultramarathon (13 females and 19 males)	Squat jump height, ankle rebound test (Leg Qindex), half squat IMVC, FVC, FEV1, PEF, MIP, 107 km race time	Cohort study	Low	MIP correlated with performance in males (*r* = 0.576, *p* = 0.010) Leg Qindex correlated with performance in females (*r* = − 0.607, *p* = 0.028)
Martinez-Navarro et al.[16]	Same cohort as above study	V̇O_2_max, VT1 and VT2, Vpeak, MFO, body composition, 107 km race time	Cohort study	Low	Correlation with race time: peak oxygen uptake (males: *r* = − 0.63, *p* = 0.004; females: *r* = − 0.85, *p* < 0.001), peak speed (males: *r* = –0.74, *p* < 0.001; females: *r* = –0.69, *p* = 0.009), speed at first (males: *r* = –0.49, *p* = 0.035; females: *r* = –0.76, *p* = 0.003) and second (males: *r* = –0.73, *p* < 0.001; females: *r* = –0.76, *p* = 0.003) ventilatory threshold, and maximal fat oxidation (males: *r* = –0.53, *p* = 0.019; females: *r* = –0.59, *p* = 0.033) Percentage of fat mass (males: *r* = 0.58, *p* = 0.010; females: *r* = 0.62, *p* = 0.024) and lean body mass (males: *r* = –0.61, *p* = 0.006; females: *r* = –0.61, *p* = 0.026) In males: Vpeak and MFO together predicted 66% of variation in race time In females: V̇O_2_max predicted 69% of variation in race time
Hoffman et al.[26]	72 athletes (17 females and 55 males) who completed a 161 km ultramarathon with qualifying criteria	BMI, BF%, 161 km race time	Cross-sectional	Low	BF% related to running speed in males (*R*^2^ = 0.23; *p* = 0.0025), but not females
Citarella et al.[27]	10 athletes (4 females and 6 males) from the Italian ultramarathon team	BMI, body composition, dietary adequacy score, training volume, record 100 km time	Cross-sectional	Low	Strong association between training volume and 100 km record time with no sex differences (ρ = 0.891, *p* = 0.009) Females had higher dietary adequacy scores than males (39.94 ± 6.33 vs. 57.50 ± 10.78; *p* = 0.038)
O’Loughlin et al.[15]	83 athletes (26 females and 57 males) who completed a 62 km trail ultramarathon	BMI, training history, pre-race experience, race time	Cross-sectional	Low	Measures associated with running performance in females: training volume (*R*^2^ = 0.116, *p* = 0.049), half marathon (*R*^2^ = 0.509, *p* = 0.0001), 10 km (*R*^2^ = 0.373, *p* = 0.021), and 5 km PB (*R*^2^ = 0.432, *p* = 0.002). In males: Age (*R*^2^ = 0.061, *p* = 0.035), BMI *R*^2^ = 0.085, *p* = 0.016), average training speed (*R*^2^ = 0.183, *p* = 0.0001), number of training years (*R*^2^ = 0.079, *p* = 0.023), marathon (*R*^2^ = 0.233, *p* = 0.002) and 5 km PB (*R*^2^ = 0.225, *p* = 0.003)
Coates et al.[29]	31 athletes (20 females and 11 males) competing in a 50 km trail race	Training and racing history, anthropometrics, BP, HR, HRV, haematocrit, CPET	Cross-sectional	Low	BMI and MAP related to performance in males (BMI: *r* = 0.75, *p* < 0.05; MAP: *r* = 0.87, *p* < 0.001), but not in females (BMI: *r* = 0.02 ns, MAP: r = -0.31 ns) Age, resting HR, HRV, V̇O_2_max and Vpeak related to 50 km performance in both sexes

IMVC, isometric maximal voluntary contraction; FVC, forced vital capacity; FEV1, forced expiratory volume over 1 s; PEF, peak expiratory flow; MIP, mean inspiratory pressure; V̇O_2_max, maximal oxygen uptake; and VT1, first ventilatory threshold; VT2, second ventilatory threshold; Vpeak, peak speed reached during cardiopulmonary exercise testing; MFO, maximal rate of fat oxidation; BMI, body mass index; BF%, body fat percentage; MAP, mean arterial pressure; PB, personal best; BP, blood pressure; HR, heart rate; HRV, heart rate variability; CPET, cardiopulmonary exercise test; and ns, not statistically significant

#### Immune Function and Oxidative Stress

Six papers reported on sex differences in immune function and/or oxidative stress in response to ultramarathons (Table 2) [30–35]. Nieman et al. found that female athletes (*n* = 9) had lower secretion rates of salivary IgA pre- and post-race, but there was no difference in the incidence of post-race upper respiratory tract infections [30]. Three papers reported on the effects of six weeks of antioxidant (AO) supplementation pre-ultramarathon [31–33]. Females were found to have higher levels of DNA damage post-race compared to males, but AO supplementation reduced this by 62% [31]. In contrast, there was no effect of AO supplementation on cellular DNA damage in males [31]. AO supplementation was found to inhibit rises in F_2_IsoPs, a marker of lipid peroxidation, in both sexes, but there were differences seen post-race in the placebo arm [33]. Male athletes’ F_2_IsoP levels remained elevated for 6 days, whereas female athletes’ levels returned to normal within 2 h. Another marker of lipid peroxidation, malondialdehyde (MDA), was significantly higher in males than females at 48 h post-race, with females demonstrating higher levels of carbonyl groups (CG), indicating greater protein oxidation [35]. Two studies looked at antioxidant repair systems and found no sex differences [34, 35]. Changes to inflammatory markers and muscle damage were also shown to be similar in males and females [32, 34].

**Table 2:** Immune function and oxidative stress

Study	Participants	Measure/s	Design	Evidence quality	Major findings
Nieman et al.[30]	31 athletes (9 females and 22 males) who finished a 160 km ultramarathon	SIgA secretion rate, Incidence of post-race URTI	Cohort study	Low	Female athletes had lower sIgA secretion rates than males both pre (358 ± 52 µg/min compared to 560 ± 38 µg/min, respectively, *p* = 0.011)- and post-race (163 ± 23 µg/min compared to 293 ± 39 µg/min, respectively, *p* = 0.008). No sex difference in post-race URTI incidence
Mastaloudis et al.[31]	22 athletes (11 females and 11 males) who completed a 50 km ultramarathon. Age 39 ± 2.5 years	Percentage of cells with DNA damage (comet assay)	RCT – double blinded	High	Females had higher levels of DNA damage post-race (gender × treatment × time interaction (*p* < 0.01) Females taking AO had 62% fewer cells with DNA damage 24 h post-race compared with placebo (*p* < 0.0008). No significant effect on males
Mastaloudis et al.[32]	Same subjects as study above	Plasma LDH and CK, Hamstrings and quadriceps MVC	RCT – double blinded	High	LDH and CK increased after the race – there was no effect of sex or AO use after correction for lean body mass. No sex differences or effect of AO on relative loss of muscle strength post-race
Mastaloudis et al.[33]	Same subjects as study above	Plasma F2-IsoPs (marker of lipid peroxidation), Plasma CRP, TNF-α, IL-6	RCT – double blinded	High	Plasma F2-IsoPs increased only in placebo group (28 ± 2 to 41 ± 3 pg/ml, *p* < .0001) In placebo group, females’ F2IsoP levels returned to normal within 2 h, whereas males’ remained elevated for 6 days post-race (gender x treatment interaction, *p* < 0.03). Inflammatory markers increased, regardless of sex or treatment group
Miyata et al.[34]	95 athletes (16 females and 79 males)	Urinary 8-hydroxydeoxyguanosine, plasma AST, CPK, myoglobin	Cohort study	Low	No sex differences in markers of oxidative stress, or antioxidant repair systems
Guerrero et al.[35]	32 athletes (13 females and 19 males)	CG (marker of protein peroxidation), MDA (marker of lipid peroxidation), GR and GPx (AO enzymes)	Cohort study	Low	48 h post-race: MDA levels were significantly higher in males (*p* < 0.05), whereas CG levels were significantly higher in females (*p* < 0.05). No sex difference in GR or GPx

sIgA, salivary immunoglobulin A secretion; URTI, upper respiratory tract infection; AO, antioxidant; DNA, deoxyribonucleic acid; RCT, randomised controlled trial; LDH, lactate dehydrogenase; CK, creatine kinase; MVC, maximal voluntary contraction; F2IsoP, F2 isoprostanes; CRP, C-reactive protein; TNF-α, tumour necrosis factor alpha; IL-6, interleukin 6; AST, aspartate transaminase; CPK, creatine phosphokinase; CG, carbonyl groups; MDA, malondialdehyde; GR, glutathione reductase; and GPx, glutathione peroxidase

#### Neuromuscular Fatigue and Cognition

Two studies investigated sex differences in neuromuscular fatigue (Table 3), with both finding that female runners demonstrate less peripheral fatigue in the plantar flexors [36, 37]. No sex differences were seen in peripheral fatigue of the knee extensors or central fatigue, although males did have higher ratings for general fatigue in races < 60 km long [36, 37].

**Table 3:** Neuromuscular fatigue and cognition

Study	Participants	Measures	Design	Evidence quality	Major findings
Temesi et al.[36]	20 athletes (10 females and 10 males) who completed a 110 km trail ultramarathon Age 44 ± 7 years	Knee extensor and plantar flexor MVC, evoked responses, fatigue and pain scores	Cohort study	Low	Females showed less peripheral fatigue in plantar flexors than males (-23% vs -8% for potentiated twitch amplitude, *p* = 0.010). No difference in knee extensor peripheral fatigue Males demonstrated greater decrease in knee extensor MVC (-38% vs -29%, *p* = 0.006). No difference in plantar flexor MVC (-26% vs -31%). No significant sex differences in measures of central fatigue
Besson et al.[37]	36 athletes (18 females and 18 males) who competed in either short (< 60 km) or long (> 100 km) ultramarathons	Knee extensor and plantar flexor MVC, evoked responses, oxygen uptake, respiratory exchange ratio (energy cost of running)	Cohort study	Low	Males had greater decrease in knee extensor MVC for all race distances (− 36% vs − 27%, *p* < 0.01). No significant difference in plantar flexor MVC Females displayed less peripheral fatigue in plantar flexors than males in races < 60 km (Δ peak twitch: − 10% vs − 24%, *p* < 0.05) Males had higher ratings for general fatigue in races < 60 km (*p* = 0.027). No sex differences in energy cost of running
Wollseiffen et al.[38]	11 athletes (6 females and 5 males)	Cognitive testing, EEG, mood state, flow state Scale-2	Cohort study	Low	No significant changes in cognitive performance, brain cortical activity, or mood states in males or females Females had higher flow ratings at pre-race (Z = − 2.22, *p* < 0.05), 3 h (*Z* = − 2.48, *p* < 0.01) and 5 h (*Z* = − 2.29, *p* < 0.05) time points

MVC, maximal voluntary contraction; and EEG, electroencephalograph

Wollseiffen et al. examined the effects of ultrarunning on cognition, brain activity, and mood and found that females reported significantly higher ratings of flow (a state of feeling fully immersed in an activity) than male runners [38]. There were no effects of ultramarathons on cognitive performance, mood, or brain activity in either sex [38].

#### Cardiopulmonary Physiology

Four studies utilised echocardiography (ECHO) to examine cardiac changes in response to ultramarathons (Table 4) [39–42]. One of these studies also employed transthoracic ultrasound to detect the presence of lung comet tails (an indicator of pulmonary oedema) and measured various blood biomarkers associated with cardiac, renal, and skeletal muscle function [42]. Concerning ECHO changes, the results were heterogeneous. One study reported no significant sex differences in the cardiac response to either a 100 km or 160 km ultramarathon [41]. In contrast, two studies involving shorter distances (55 km or 70 km) reported a lower incidence of ECHO changes indicative of cardiac fatigue in females [39, 40]. Tiller et al. measured stroke volume and cardiac output before and after a 171 km race and reported the only significant change to be an increase in cardiac output in females [42].

**Table 4:** Cardiopulmonary physiology

Study	Participants	Measures	Design	Evidence quality	Major findings
Picco et al.[39]	28 athletes (11 females and 17 males) who completed either a 70 km or 55 km mountain ultramarathon Age 38 ± 9 years	ECHO: EF, GLS, RVFWS, ventricular torsion and volume	Cohort study	Low	Whole cohort: Significant post-race decreases in EF (62.7 ± 6.1% to 57.2 ± 8.7%, *p* = 0.008), GLS, RVWS, LA volume and LV end diastolic dimensions. Post-race increases in RA area and RV diastolic dimensions. Females: only reduced LV dimension (*p* = 0.05) and increased RV diameter (*p* = 0.012) were significant
Picco et al.[40]	24 athletes (5 females aged 38 ± 4 years, and 19 males aged 42 ± 12 years) who completed a 55 km mountain ultramarathon	ECHO, serum lactate and CPK	Cohort study	Low	Females had lower incidence of ECHO features of exercise induced cardiac fatigue for the right ventricle (*p* = 0.3) and the left ventricle (*p* < 0.001) Females had significantly lower serum lactate post-race (2.63 mmol/L vs 4.37 mmol/L, *p* = 0.018). No difference in CPK
Cote et al.[41]	25 athletes (8 females aged 45.9 ± 10.2 years, and 17 males aged 44.8 ± 6.6 years) who completed either a 100 km or 160 km ultramarathon	ECHO	Cohort study	Low	No significant sex differences in the cardiac response to an ultramarathon
Tiller et al.[42]	16 athletes (8 females and 8 performance-matched males) who completed either the UTMB or CCC races in 2018 or 2019. Average age 38.4 ± 7.6 years. Average finish time (hh:mm) 30:52 ± 10:42	Vital signs, ECG, anthropometry Serum electrolytes, Hb concentration, Hct, and biomarkers Pulmonary function tests. Resting lung diffusing capacity, transthoracic ultrasound	Cohort study	Low	Females and males exhibited significant pre- to post-race increases in BNP (25.8 ± 14.6 vs. 140.9 ± 102.7 pg/mL, *p* = 0.007; and 26.6 ± 17.5 vs. 96.4 ± 51.9 pg/mL; *p* = 0.002, respectively) and CK-MB (3.3 ± 2.4 vs. 74.6 ± 49.6 IU/L, *p* = 0.005; and 7.2 ± 3.9 vs. 108.8 ± 37.4 IU/L; *p* = 0.002, respectively), while males only exhibited significant pre- to post-race increases in Cr (1.06 ± 0.19 vs. 1.23 ± 0.24 mg/dL; *p* = 0.028). Females exhibited significant pre- to post-race decreases in FVC (*p* = 0.008, d = 0.79), PEF (*p* = 0.039, d = 0.92), IC (*p* = 0.004, d = 1.46), Fe_NO_ (*p* = 0.031, d = 0.66), and P_IMAX_ (*p* = 0.028, d = 0.47), while males exhibited significant pre- to post-race decreases in PEF (*p* = 0.048. d = 0.25), IC (*p* = 0.005, d = 0.79), Fe_NO_ (*p* = 0.038, d = 0.97), DL_CO_ (*p* = 0.004, d = 0.83), DL_NO_ (*p* = 0.002, d = 0.88), and V_C_ (*p* = 0.002, d = 1.22) Both males and females exhibited significant pre- to post-increases in lung comet tails (*p* = 0.006, d = 2.41; and *p* = 0.048, d = 0.96, respectively). Pooled effect size was greater in males (*d* = 0.86) than in females (*d* = 0.63)
Martinez-Navarro et al.[43]	32 athletes (13 females and 19 males) who completed a 107 km mountain ultramarathon	Spirometry, peak expiratory flow rate (PEF)	Cohort study	Low	No significant sex differences in spirometry changes during and post ultramarathon aside from significantly greater decline in FEV1/FVC in females (16.1% vs 3.5%, *p* = 0.019)

EF, ejection fraction; GLS, global longitudinal strain; RVFWS, right ventricular free wall strain; LA, left atrium; LV, left ventricle; RA, right atrium; RV, right ventricle; CPK, creatine phosphokinase; ECHO, echocardiography; PEF, peak expiratory flow; FEV1/FVC, ratio of forced expiratory volume in 1 s to the forced vital capacity; UTMB, Ultra-Trail du Mont-Blanc; CCC, Courmayeur–Champex–Chamonix; ECG, electrocardiography; Hb, haemoglobin; Hct, haematocrit; BNP, brain natriuretic peptide; CK-MB, creatine kinase-MB isoenzyme; Cr, creatinine; PV, plasma volume; FVC, forced vital capacity; IC, inspiratory capacity; Fe_NO_, exhaled nitric oxide; P_IMAX_, maximum static inspiratory pressure; DL_CO_, diffusing capacity for carbon monoxide; DL_NO_, diffusing capacity for nitric oxide; and V_C_, pulmonary capillary blood volume

This study investigated the frequency of pre- to post-race physiological perturbations in race time-matched males and females and found that participation in ultramarathon resulted in biomarker derangement, decrements in pulmonary function, and the development of lung comet tails on ultrasound in both groups. However, only the male group demonstrated a statistically significant reduction in lung diffusing capacities and pulmonary capillary blood volume [42]. Additionally, the frequency and effect size of alterations in biomarkers and lung comet tails were smaller in the female cohort [42]. Martinez-Navarro et al. used spirometry to assess pulmonary function throughout a 107 km mountain ultramarathon and found the only measure differing between males and females to be a greater decline in FEV1/FVC ratio in females [43].

#### Other Physiology

Three papers reported on physiological parameters outside of the sub-topics covered above (Table 5) [45–47]. One looked at changes in creatine kinase (CK) and the relationship between CK and sodium following a 161 km ultramarathon and found no significant sex differences [45]. Another study measured biomarkers of liver injury following a similarly long ultramarathon and also found no difference between male and female runners [46]. The third study, involving a 90 km road ultramarathon, looked at a variety of physiological markers and reported that females sustained a higher fraction of their V̇O_2_max throughout the race, and had significantly lower plasma free fatty acids (FFA) post-race compared with males [47]. There were no significant sex differences in serum glucose or osmolality [47].

**Table 5:** Other physiological measures

Study	Participants	Measures	Design	Evidence quality	Major findings
Hoffman et al.[45]	216 athletes (40 females and 176 males) aged 42.6 ± 9.4 years who completed a 161 km ultramarathon	Serum CK and sodium	Cross-sectional	Low	No significant sex differences in serum CK. Sex differences in sodium not reported
Speechly et al.[47]	20 athletes (10 females aged 33.6 ± 5.6 years, and 10 males aged 35 ± 8.8 years). Marathon performance-matched pairs signed up to a 90 km road ultramarathon	90 km race time, V̇O_2_max, running economy, serum glucose, osmolality, FFA	Cohort study	Low	Females had significantly faster average running speed during 90 km ultramarathon than males (171 m/min vs 155.2 m/min, *p* < 0.05) and sustained a higher fraction of their V̇O_2_max during the ultramarathon (59.8 ± 6.2% vs 50.2 ± 3.1%, *p* < 0.01) Females had significantly lower plasma FFA post-race (*p* < 0.01). No sex differences in serum glucose, serum osmolality, or running economy
Tirabassi et al.[46]	36 athletes (8 females and 28 males) aged 43 ± 10 years who completed a 100 mile ultramarathon at altitude	Serum liver enzymes, CK, bilirubin	Cohort study	Low	No significant sex differences in biomarkers of liver injury post-race

CK, creatine kinase; V̇O_2_max, maximal oxygen uptake; and FFA, free fatty acids

#### Chronic Health Issues/Lifestyle Factors

Four studies examined the sex differences in chronic health and lifestyle factors affecting ultramarathon runners (Table 6) [48–51]. Female ultrarunners were found to have higher rates of hypothyroidism and sleep disorders and were more likely to take supplements [48, 49]. When compared to the general population, female runners were shown to have more regular bowel motions [50]. A negative correlation was found between training volume and ferritin levels in both males and females [50]. Although female ultrarunners were more likely to have a history of bone stress injury, a greater proportion of males returned Z scores < 1 following DEXA scanning [51]. Female ultrarunners were more likely to have a BMI < 18.5 kg/m^2^, an elevated risk of eating disorders, and a moderate triad cumulative risk score. There was no difference in the percentage of males and females returning a high-risk score on this measure [51].

**Table 6:** Chronic health issues/lifestyle factors

Study	Participants	Measures	Design	Evidence quality	Major findings
Boldt et al.[48]	281 athletes (159 females aged 37.7 ± 10.5 years, and 122 males aged 42.8 ± 11.1 years) including a 10 km control group, 103 half marathoners and 70 marathon/ultramarathon runners	Health survey	Cross-sectional	Low	Higher rates of hypothyroidism in females (X^2^ = 8.515, *p* = 0.014, φc = 0.174). Females more likely to take supplements prescribed by a doctor (X^2^ = 8.554, *p* = 0.014, φc = 0.174). Males more likely to report weight loss resulting from running (X^2^ = 9.444, p = 0.024, φc = 0.183),
Tokudome et al.[50]	180 athletes (36 females aged 48.9 ± 6.9 years, and 144 males aged 50.5 ± 9.4 years) entered in a 2 day ultramarathon	Health survey, blood indices, BMI	Cross-sectional	Low	Training volume was negatively correlated with ferritin in both sexes. Female runners were more likely to report daily bowel motion than the general population (96.5%, 95% CI 92–100%; vs 70.5%, 95% CI 68.4–72.5%)
Martin et al.[49]	636 athletes (95 females and 541 males). From Italy, France and United States	Sleep survey	Cross-sectional	Low	Prevalence of reported sleep disorders was 38.9% in females compared with 22% in males (*p* < 0.005)
Hoeg et al.[51]	123 athletes (40 females and 83 males). Mean age 41.8 and 46.2 years, respectively) who competed in a 100 mile ultramarathon in 2018 or 2019	Triad cumulative risk assessment score, DEXA, serum ferritin, vitamin D, sex hormones	Cross-sectional	Low	Proportion of athletes with: elevated risk of eating disorders: Males 44.5%, females 62.5%; history of bone stress injury: Males 20.5%, females 37.5%; BMI < 18.5 kg/m^2^: Males 0%, females 15%; BMD Z score < 1.0: Males: 30.1%, females 16.7%; Triad cumulative risk assessment: males—29.2% moderate risk, 5.6% high risk; females—61.1% moderate risk, 5.6% high risk

BMI, body mass index; DEXA, dual-energy X-ray absorptiometry; BMD, bone mineral density; and CI, confidence interval

#### Training or Race-Related Illness and Injury

Of the twelve studies examining illness and injury incurred as a direct result of running, eleven measured rates over one event, and one looked at injury incidence across 12 months (Table 7) [52–63]. There was no difference in the overall rate of exercise-related injuries across a 12-month period; however, female athletes suffered a disproportionate number of stress fractures [52]. Females were more likely to encounter medical illness during a race, with one study demonstrating four times the risk of developing an AKI during a multi-stage ultramarathon compared to males [53, 54]. One study found that males were significantly more likely to report a history of heat-related illness and muscle cramps, and to experience heat-related symptoms during an ultramarathon [55]. However, another study reported similar rates of exercise-associated muscle cramps in males and females [56]. When overall race-related injury and illness rates were examined, there was no significant difference between the sexes [53, 57].

**Table 7:** Training or race-related illness and injury

Study	Participants	Measures		Design		Evidence quality		Major findings
Hoffman & Krishnan [52]	1,212 active ultramarathon athletes (32% female, 68% male). Median age 42.3 years (range 18–81 years)	12-month incidence of exercise-related injury and stress fractures		Cohort study		Low		No sex differences in rate of exercise-related injuries over 12-month period. Stress fractures were more common among females, compared with males (*p* < 0.001)
Khodaee et al (Abstract only) [57]	308 athletes who completed a 100 mile ultramarathon. Sex split not reported	Race-related injuries requiring medical attention post-race		Cross-sectional		Low		No sex differences in incidence of race-related injuries requiring medical attention
Krabak et al.[53]	396 athletes (20.8% females, 79.2% males) who completed a 250 km 7-day trail ultramarathon	Rate of medical illness and injury		Cohort study		Low		Females were 16% more likely to encounter medical illness when adjusted for age and race hours No difference in overall injury/illness rates, skin problems, or MSK injuries
Lipman et al.[54]	128 athletes (28% females and 72% males) who competed in a 250 km multi-stage ultramarathon	Changes in serum creatinine, cumulative incidence and prevalence of AKI		Cohort study		Low		Overall cumulative incidence of AKI was 41.4%. Odds ratio for females and AKI was 4.64 (95% CI 2.07–10.37, *p* < 0.001)
Hoffman et al.[58]	201 athletes who completed a 161 km ultramarathon. Sex split not reported	Plasma sodium, incidence of EAH		Cohort study		Low		Overall EAH incidence was 6%. No sex differences in incidence of EAH
Rust et al.[59]	46 athletes (19 females and 27 males) who completed a 100 km ultramarathon	Body mass, plasma and urinary electrolytes, Hct, plasma volume, fluid, energy, and electrolyte intake		Cohort study		Low		5% (n = 1) of females and 11% of males (n = 3) developed EAH. Significant decrease in Hct in females (-1.2%, p > 0.05) but not in males (-1.1%, *p* < 0.05). No sex differences in electrolyte changes
Hoffman et al.[62]	47 athletes (Sex split not reported) who completed a 161 km ultramarathon	Serum sodium		Cohort study		Low		Overall EAH incidence was 30% Females made up 28.6% of hyponatraemic group and 18.2% of normonatraemic group (significant > 0.05)
Chlibkova et al.[60]	113 athletes (25 females and 88 males) who completed an ultramarathon		Serum sodium	Cohort study	Low		No sex differences in the incidence of hyponatraemia	
Costa et al.[61]	74 athletes (28 females and 46 males) who completed a 225 km multi-stage ultramarathon Mean age 41 years		Fluid and sodium intake, serum sodium and osmolality, plasma volume, urine osmolality	Cohort study	Low		Females had a higher water intake (daily and during running) when corrected for body mass (*p* < 0.001). No significant sex differences in sodium intake, serum sodium, plasma volume, serum osmolality	
Khodaee et al.[63]	84 athletes (15 females and 69 males) who competed in a 100 mile high altitude ultramarathon in Colorado		Serum sodium, post-race survey	Cohort study	Low		15.9% of males had EAH, compared with 40% of females (*p* = 0.66)	
Schwellnus et al.[56]	49 athletes who completed a 56 km ultramarathon		Incidence of EAMC	Cohort study	Low		No sex differences of EAMC incidence during, or within 6 h of an ultramarathon	
Bouscaren et al.[55]	3126 athletes (525 females and 2601 males) who competed in a trail ultramarathon on Reunion Island. Mean age 42.2 years		Pre- and post-race survey	Cohort study	Low		History of HRI: 79.9% of males vs 70.2% of females (*p* < 0.001) History of muscle cramps: 40.9% of males vs 22.5% of females (*p* < 0.001). No significant sex differences in history of digestive issues, headaches, or collapse Experienced heat-related symptoms during race: 56.5% of males vs 46.8% of females (*p* = 0.002)	

MSK, musculoskeletal; AKI, acute kidney injury; Hct, haematocrit; EAH, exercise-associated hyponatraemia; HRI, heat-related illness; and EAMC, exercise-associated muscle cramps

Six studies investigated sex differences in sodium and fluid balance during ultramarathons [58–63]. Despite females having a higher water intake in one study, and a significant decrease in haematocrit in another, no statistically significant differences in hyponatraemia incidence were found [58–63].

#### Pacing Strategy

There were conflicting findings among the 6 studies investigating pacing strategies (Table 8) [9, 64–68]. Three studies found no significant sex differences in the pacing strategies employed during ultramarathons [65–67]. Two studies reported more even pacing in females, while the final study found higher pace variation among the female athletes [9, 64, 68]. Despite these heterogeneous results, the studies were in agreement regarding the benefit of more even pacing, which was shown to be associated with better race times in both sexes [9, 64–68].

**Table 8:** Pacing

Study	Participants	Measures	Design	Evidence quality	Major findings
Suter et al.[9]	13,829 athletes (1148 females and 12,681 males) who completed the UTMB between 2008–2019	Average running speed, pace variation	Retrospective data analysis	Low	More even pacing was associated with better finish times in males and females Females demonstrated higher pace variation than males
Bossi et al.[65]	501 athletes (103 females and 398 males) who competed in a 24 h ultramarathon held in Brazil	Mean running speed for each hour	Cohort study	Low	Both sexes demonstrated a reverse J-shaped pacing pattern, with no significant difference between sexes. More even pacing was associated with better performances in males and females
Moffit & Call [68]	1,453 athletes (462 females and 991 males) who completed a 100 mile race held in the United States between 2010–2019	Running pace, %MRS	Retrospective data analysis	Low	Females ran at a significantly lower %MRS in the first third of the race and finished with a significantly higher %MRS than males. A more even pacing strategy was associated with better performances in males and females
Inoue et al.[66]	51 athletes (21 females and 30 males) who completed a 24 h track ultramarathon held in Brazil	Time per 400 m, time spent in SSR	Cohort study	Low	Both sexes demonstrated a reverse J-shaped pacing pattern. In both sexes, high-performance runners spent significantly less time in SSR than low-performance runners
Renfree et al.[64]	196 athletes (57 females and 139 males) who competed in the 100 km World Masters Championship	Relative speed per 10 km segment, pace CV	Cohort study	Low	Males displayed significantly higher relative speeds in the first (*p* = 0.03), second (*p* < 0.01), and third (*p* < 0.01) 10 km segments. Females had significantly higher relative speeds in the ninth segment (*p* = 0.01) CV over intermediate segments were 7.39% for females and 10.33% for males
Deusch et al.[67]	937 athletes (260 females aged 46.83 ± 12.33 years, and 677 males aged 49.3 ± 11.52 years) who completed one of 12 time-limited ultramarathons on flat asphalt in Europe	CV of time per lap, average running speed	Retrospective data analysis	Low	There was no main effect of sex on pacing. Both sexes demonstrated a higher CV for 24-h races compared with 6- and 12-hour races

UTMB, Ultra-Trail du Mont-Blanc; %MRS, percentage of mean running speed; SSR, significant speed reduction; and CV, coefficient of variability

## Discussion

This purpose of this review was to determine if significant sex differences in ultramarathon runners exist, which could justify the development of separate guidelines for males and females. While the overall quality of evidence from the included studies is low, by pooling the results, coherence in some areas has emerged. Evidence of sex differences in ultramarathon runners was identified in the predictors of performance, the physiological responses to racing, and the risk of acute and chronic illness and injury. Areas where no sex differences were found included cognitive performance, central fatigue, and overall injury rates. However, given the predominance of low-quality evidence, confidence in this evidence is also low, and these results should be interpreted with caution. Presently, a personalised approach based on the individual’s responses to training, racing, nutrition, and recovery interventions is recommended.

### Areas Where Evidence of Sex Differences Exists

See Fig. 2.

**Fig. 2:**
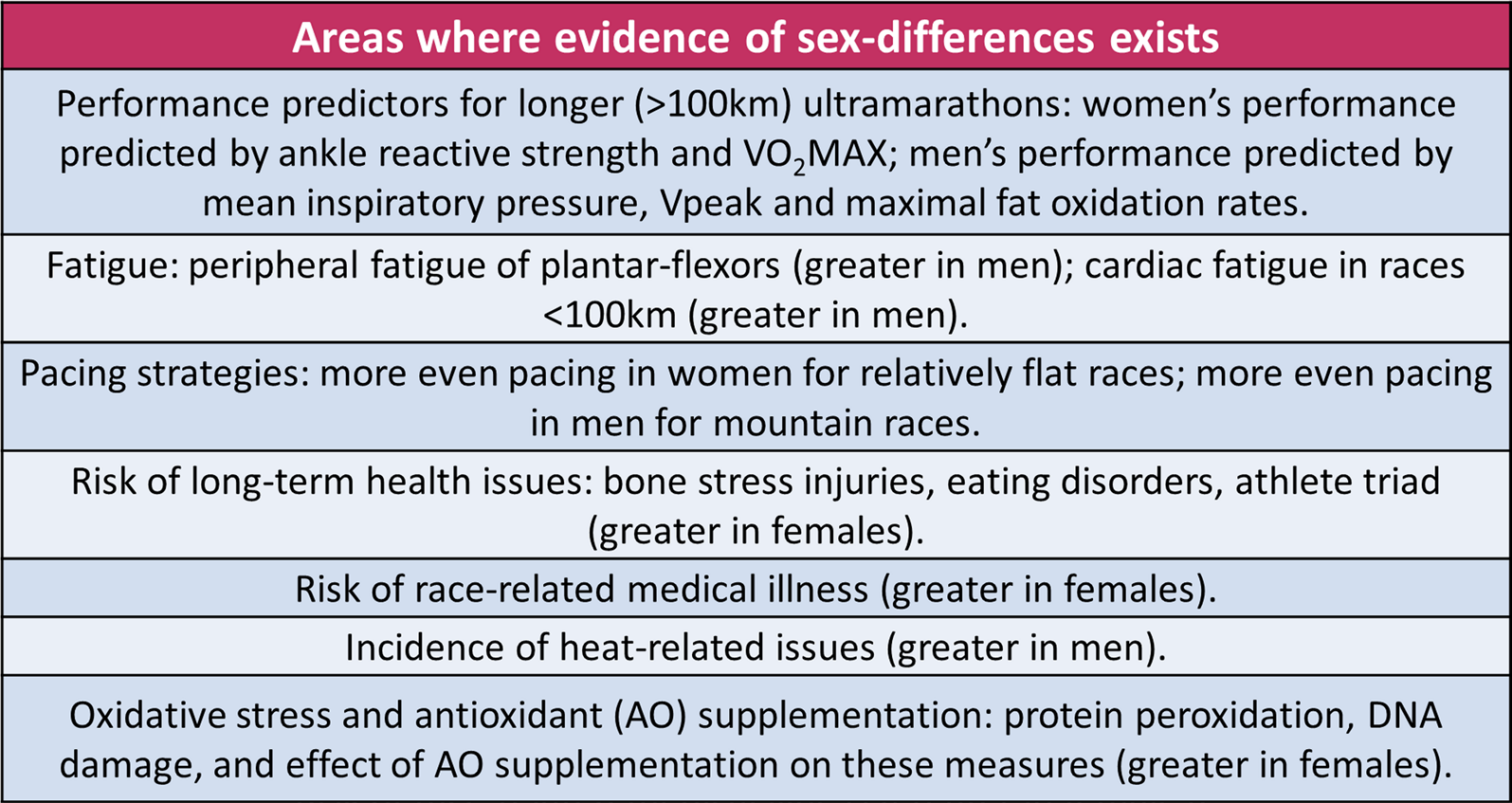
Areas where evidence of sex differences exists

#### Predictors of Performance

There is reasonable evidence that ultramarathon performance is differently predicted in males and females [15, 16, 26–29]. This is important as it could translate into different training priorities for each sex. For example, a strong correlation between ankle rebound performance and female ultramarathon performance was demonstrated by Martinez-Navarro et al. [28] Conversely, mean inspiratory pressure was correlated to performance in males only [28]. Indeed, there is evidence that females have superior fatigue resistance of the respiratory muscles, indicating that this may be less of a performance limiting factor than in males [69]. While these findings may imply that males and females would benefit from targeting different variables in training, this is a single study with a small sample size. Furthermore, the method employed for measuring inspiratory pressure is largely dependent on the effort imparted by the subject and these results may therefore represent differences in motivation, rather than respiratory muscle strength and fatiguability. Therefore, larger studies utilising respiratory muscle nerve stimulation techniques are required before conclusions can be drawn.

Significant sex differences were also found when anthropomorphic variables were examined [15, 26, 27, 29]. Several studies found that BMI and body composition correlated with ultramarathon performance in males, but not in females [15, 26, 29]. Furthermore, while Martinez-Navarro et al. found that body composition correlated to performance in both sexes, this relationship did not persist after multiple regression analysis [16]. Conversely, much larger studies of runners engaging in races of half marathon distance and below have found that body composition is predictive of performance in males and females [70, 71]. Thus, it is possible that the association between body composition and performance becomes less important for female runners as race length increases. However, given the small sample sizes and the high probability that females participating in studies of extreme endurance events are not representative of the wider female ultrarunning community, it is not possible to draw conclusions at this time.

The classic physiological measures of fitness that predict performance also differ between males and females. In one study of runners completing a 107 km race, performance was independently predicted by MFO and Vpeak in males, but only by V̇O_2_max in females [16]. This implies that high-intensity training targeting V̇O_2_max improvements could optimise female performance, whereas males may benefit from more sustained efforts to improve fatigue resistance and fat oxidation rates. However, as these studies only provide evidence of correlation of these physiological measures and performance, this hypothesis needs to be tested. Studies investigating the sex differences in response to varying ultramarathon training stimuli would provide useful and practical insights for ultramarathon coaches and their athletes.

In summary, there is currently low-level evidence that male and female ultramarathon performance is predicted by different variables, but this area of research needs significant development before these findings can be used to guide training priorities.

#### Fatigue

During demanding physical pursuits such as ultramarathons, fatigue affects many body systems and can strongly influence performance [72]. This review found evidence that females experience less peripheral and cardiac fatigue than males in ultramarathons, which could confer an advantage in longer races, and influence training plan design [36, 37, 39, 40]. Indeed, the performance gap between males and females does appear to decrease with increasing race length [73]. This review included two small cohort studies which examined muscle fatigue in relative performance-matched, female and male ultramarathon runners. The female runners demonstrated less peripheral fatigue of the plantar flexors and smaller decrements in knee extensor maximal voluntary contraction (MVC) following ultramarathon races [36, 37]. This is in agreement with studies in non-athlete populations, and could be due to several factors including differences in muscle fibre type, and less accumulation of anaerobic metabolites involved in inhibitory feedback loops [2, 17, 74, 75].

Females also demonstrated reduced exercise-induced cardiac fatigue (EICF) in races under 100 km [39, 40]. EICF is characterised by a reduction in left ventricular systolic and diastolic function following prolonged, strenuous activity [41]. A propensity for less EICF could not only imply a female advantage in ultramarathon racing, but could also have implications for the long-term cardiac effects of ultramarathon training [76]. The reason for this relative fatigue resistance in females is not known; however, given the protective effects of oestrogen on the myocardium, it is likely that sex hormones play a role [76]. This could be investigated by comparing EICF in post-menopausal and premenopausal females in future studies.

It should be noted that an alternative explanation for the apparent superior “fatigue resistance” of female athletes exists. It has been suggested that male and female athletes approach ultramarathon races with different competition intentions which could explain the greater fatigue often reported in males [17]. For example, in Besson et al.’s study, females reported greater motivation to enjoy races < 60 km, whereas males were more competitively oriented, although no sex differences were found for races > 100 km [17]. This competitive orientation could result in males tapping into their “security reserves” more than females, resulting in greater decrements in force production capacities.[17]. Indeed, a survey of 344 female recreational ultramarathon runners found that females rated personal achievement and physical health as greater motivators than competition [77]. However, it is likely that motivational factors also vary significantly between recreational “weekend warriors” and top ultramarathon athletes competing for prize money [78]. Regardless of whether physiological or motivational factors underlie the findings, the examined literature suggests that males experience more peripheral and cardiac fatigue than females which could impact the design of training plans with regard to planning recovery.

#### Pacing

It is possible that interactions exist between the aforementioned relative fatigue resistance in females, and pacing strategies utilised during races [2]. This could provide useful information for coaches and athletes planning racing strategies. Tiller et al. postulate that females may be better able to maintain running pace as a consequence of less peripheral muscular fatigue [2]. However, the findings of this review were mixed. While three studies looking at 24 h time-limited marathons failed to find sex differences in pacing strategies, females demonstrated more even pacing over the 100 km World Masters Championship race and a relatively flat 100 mile race [64–68]. Conversely, females had greater pace variation during the Ultra-Trail du Mont-Blanc, a mountain ultramarathon involving 10,000 m vertical gain [9]. Given that males have greater muscle mass relative to fat mass, it has been suggested that they may be better able to maintain pace going uphill and hence have less pace variation on mountainous terrain [9]. However, the link between body composition and pacing remains speculative at this stage, and the relationship between race format and terrain and sex differences in pacing requires further examination.

#### Health

As with the wider population, the health concerns most commonly affecting athletes can differ according to sex [79]. This review found this to be true in ultramarathon runners. For example, female ultramarathon runners were found to report higher rates of sleep disorders, have greater rates of bone stress injuries, and be more at risk of eating disorders [49–51]. These findings should be considered when designing training and recovery programmes for females.

Sleep plays an important role in performance, injury rates, inflammation, and recovery [49]. While only one study in this review examined sleep disorder prevalence, it had a reasonably sized female cohort (*n* = 95) and the findings are supported by larger studies in both ultramarathon runners and general populations [49, 80–82]. A meta-analysis of 29 studies found that females were 40% more likely to suffer from insomnia than males [82]. However, these studies largely relied on self-reported measures of sleep. A recent paper on sleep in junior endurance athletes found that females had worse subjective sleep quality, but objectively achieved higher total sleep time and greater sleep efficiency [83]. Thus, it is unclear if the preponderance of self-reported sleep disorders in female ultrarunners represents an objectively worse quality of sleep which could impact training and recovery. Interestingly, in the aforementioned study, objective sleep quality was influenced by different stages of the menstrual cycle, which could indicate variable recovery requirements for female athletes across the cycle [83]. This premise needs further development, and studies on the effect of the menstrual cycle and sleep in ultramarathon runners do not yet exist. However, given the widely acknowledged important role sleep plays in athletic recovery, it is reasonable to conclude that all ultramarathon runners, irrespective of sex, would benefit from interventions to improve sleep [84].

The greater rate of bone stress injuries (BSIs) in female ultrarunners is significant because injuries that relate to poor bone health are associated with osteoporosis and fragility fractures in older age [51, 52]. Moreover, female ultramarathon runners have an elevated risk of eating disorders and the athlete triad [51]. While low energy availability negatively affects all athletes, the consequences for females are more rapid, and even within-day deficits affect menstrual function and bone turnover [21, 85]. Interestingly, in the study by Høeg et al., male ultramarathon athletes were in fact more likely to have low bone mineral density than females, and further research is required to confirm this finding [51]. The same study found that over half of the female cohort, and none of the males, had below normal levels of serum 25-hydroxyvitamin D [51]. There is evidence that vitamin D supplementation may be protective against BSIs in athletic populations, and a recent review has suggested this approach in athletes with low serum levels [17]. Professionals working with female athletes should be aware of these findings, as a failure to address disordered eating practices, and/or the prescription of excessive training loads, could have severe short- and long-term effects on performance and health. Additionally, assessing serum 25-hydroxyvitamin D levels and supplementing those who are deficient appears to be especially important for female ultrarunners.

The studies included in this review found that, during multi-day ultramarathons, female runners were more likely to encounter a medical illness, and one study identified a four-fold increase in rates of acute kidney injury (AKI) [53, 54]. The reasons for these findings are not currently known, and further studies are needed to confirm this female propensity for illness and clarify the nature of illnesses encountered. Furthermore, while increases in serum creatinine (such as in AKI) are extremely common following an ultramarathon, renal function rapidly normalises in most individuals without clinical consequence [7, 86]. However, hospitalisations for renal failure, while rare, do occur among ultramarathon runners, and the long-term effects of repeated insults to renal function in this setting are not yet known [87]. It is therefore unclear what relevance this finding has for female ultramarathon runners.

Ultramarathons often take place in challenging environments, including extreme heat, and sex differences have been found in issues such as heat-related illness and muscle cramps [88]. Understanding an athlete’s ability to train and perform in the heat may be important for choosing goal races and planning training schedules and heat acclimatisation strategies. This review included two studies examining this issue. A study of 49 ultramarathon runners found no association between sex and the incidence of muscle cramping during a 56 km road race in Cape Town [56]. In contrast, a larger study of over 3000 athletes competing in a trail ultramarathon on Reunion Island found a higher incidence of heat-related symptoms and muscle cramps in males [55]. These disparate results could be due to the different climates in which the studied races took place, with Reunion Island having significantly higher average temperatures than Cape Town in the months that the races were held. It is also possible that the first study was underpowered to detect a difference given the smaller subject numbers. Females have demonstrated more efficient heat dissipation via sweat evaporation than males, and a greater ability to maintain their core temperature in hot and humid environments [88, 89, 91]. However, these data are not specific to ultraendurance exercise and research in ultramarathon runners is required to determine if these findings hold true under the conditions experienced by this population.

Ultrarunning is a significant source of oxidative stress, and there is robust evidence of increased reactive oxygen species (ROS) and free radicals following ultramarathon races [31, 35]. ROS are thought to regulate acute-phase inflammatory responses, and could therefore affect ultramarathon performance and recovery [35, 92]. This review found that the oxidative response to ultramarathons is influenced by sex, with females demonstrating significantly higher levels of protein peroxidation indicators and cellular DNA damage following an ultramarathon [35, 36]. Moreover, females showed a greater response to antioxidant supplementation on minimising DNA damage [35]. While this may seem beneficial, studies have shown that long-term AO supplementation can impair adaptation to endurance training [93–95]. Unfortunately, most of these studies have been carried out on male subjects. However, one study included only female runners and found that vitamin C supplementation was associated with a slowing of running speeds during training, although no effect on 5 km time trial performance was seen [96]. Thus, while AO supplementation may have a greater effect on reducing oxidative stress in females, the implications of this are unclear [97]. More studies examining the long-term effects of AO supplementation on health and performance are required before conclusions can be drawn.

### Areas Where There Is No Evidence of Sex Differences

See Fig. 3.

**Fig. 3:**
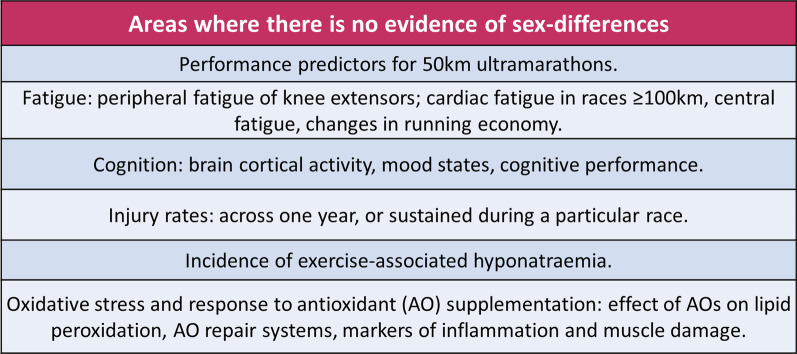
Areas with no evidence of sex-differences

#### Predictors of Performance

While most studies in this review found sex differences in the predictors of ultramarathon performance, there were several areas where male and female performance was similarly predicted. For example, while Martinez-Navarro et al. found that V̇O_2_max was predictive of female 107 km ultramarathon performance only, in a shorter 50 km race, V̇O_2_max and Vpeak were related to performance in both sexes [16, 29]. Given the significant difference in the lengths of these races, these conflicting findings are perhaps unsurprising and could be due to physiological demands changing as race length increases. For example, females may have a lesser propensity to experience fatigue of the myocardium, respiratory musculature, and skeletal muscles, and these factors could be less limiting to performance compared with males [36, 37, 39, 40, 69]. Consequently, maximal oxygen uptake may become less important for males as distance increases and other systems fatigue. Further studies looking at sex differences over a variety of race distances are needed to clarify how the physiological predictors of performance relate to race duration.

Training volume is another predictor of performance which is potentially shared by the sexes, but the research on this is inconclusive [27]. While Citarella et al. found a strong association between training volume and record 100 km time in males and females, their sample consisted of only 10 elite athletes [27]. In contrast, O’Loughlin et al. studied recreational athletes running a 62 km ultramarathon and found that training volume was predictive of female performance only [15]. It is likely that the influence of training volume on performance varies according to race distance. Additionally, it is possible that different variables predict performance in elite runners, compared with recreational runners, and this is another area which warrants further investigation.

#### Fatigue

While females are considered more fatigue-resistant than males in general, this review found several areas where no sex difference was found. Firstly, there were no differences in measurements of central fatigue, brain cortical activity, mood states, and cognitive performance both during and after ultramarathon participation [36, 38]. Secondly, although males demonstrated greater peripheral fatigue of the plantar flexors, there were no sex differences in peripheral fatigue of the knee extensors [36, 37]. Temesi et al. postulate that this discrepancy may be explained by greater Achilles tendon compliance in females, which has been associated with superior maintenance of work output and greater mechanical efficiency of the soleus in rat models [36]. However, this association has not been replicated in human subjects and remains speculative at this stage. Additionally, despite evidence of greater decrements in contractile function in males relative to females, this did not translate into differences in the energy cost of running [37]. This may be because the sex differences in neuromuscular fatigue are not large enough to affect running economy [37]. It is therefore unclear what relevance these findings have for ultramarathon training and, as only one study looked at this relationship, these findings require confirmation.

Lastly, while sex differences in EICF were demonstrated in shorter ultramarathon races, none were found in races of 100 km and 160 km [39–41]. This could be related to the lower exercise intensity that is maintained in longer races. However, due to the small female cohorts studied, this research should be interpreted with caution. Larger studies including intensity-matched males and females are required to further investigate sex differences in EICF. Furthermore, research into the long-term consequences of repeated bouts of EICF is required before the relevance of these findings can be discussed further.

#### Health

Similar overall injury rates were found in males and females by all three studies examining the topic [52, 53, 57]. One study found that approximately 65% of ultramarathon runners had experienced a running-related injury in the preceding 12 months [52]. The other two studies examined injuries encountered during a race and found no association between sex and musculoskeletal, or skin-related problems [53, 57]. These findings are in agreement with research into runners in general. A recent meta-analysis found that while females are more likely to sustain BSIs, and males are more likely to be affected by Achilles tendinopathies, overall injury rates are the same [98].

Female sex has been reported as a risk factor for exercise-associated hyponatraemia (EAH), which most commonly results from overconsumption of hypotonic fluids [97]. However, where studies on ultramarathon runners are concerned, the majority failed to find a significant increase in the rates of EAH in females [58–60]. Two studies reported higher rates in females which failed to reach statistical significance, and one study actually demonstrated higher rates in males [59, 61, 62]. Thus, it seems unlikely that female ultramarathon runners are at a significantly greater risk of EAH. Nevertheless, there is a risk of over-drinking during races if females are not given individualised hydration guidelines based on body weight and sweat rates. Additionally, there are significant fluctuations in core temperature across the menstrual cycle, and elevations in oestradiol and progesterone are associated with fluid retention and greater sodium loss [13, 98]. These cyclical changes could confound the data, and further research on the effects of sex on sodium balance should account for hormonal fluctuations.

In terms of oxidative stress resulting from ultramarathon racing, AO supplementation effectively prevented rises in markers of lipid peroxidation in both sexes, and there were no differences found in antioxidant repair systems [31, 33]. Furthermore, when looking at markers of inflammation and muscle damage following ultramarathons, either with or without AO supplementation, no sex differences were found [33, 45]. Therefore, the significance of sex similarities and differences in oxidative stress is unclear at present.

## Limitations

There were several limitations to this review. Firstly, the search criteria excluded studies involving other endurance sports and running events of marathon length and under. While this was done to increase the specificity of findings to the sport of ultramarathons, it is possible that relevant findings from other disciplines were overlooked. Secondly, although representation of both sexes in cohorts was an inclusion criterion, females were still outnumbered by males, sometimes considerably, which could affect the validity of some of the findings. Thirdly, fluctuations in sex hormones across the menstrual cycle have important implications for many of the reported outcomes; however, this was rarely accounted for in the included studies. Additionally, the term “ultramarathon” includes races involving a vast range of distances, formats, and environments. Thus, caution must be used when extrapolating findings from studies based on one race, to ultramarathons as a whole. Finally, this review was conducted by one researcher and therefore did not benefit from duplicate data extraction and multiple independent reviewers, which could have affected the reliability of the search.

## Conclusion

This review has demonstrated that although the importance of sex-related differences in physiology is garnering increasing recognition, significant gaps in knowledge around the extent and significance of these differences remain. As reflected by the results, there are very few interventional studies comparing male and female ultrarunners, and observational studies predominate. Furthermore, female sample sizes are generally small and may not be representative of the broad range of ultramarathon participants. Thus, the evidence base is generally of poor quality, making it difficult to infer conclusions regarding the potential benefits of sex-specific approaches to ultramarathon training, racing, nutrition, and recovery. Additionally, due to the wide range of ultramarathon race formats, significant heterogeneity in the race distance and terrain exists between studies, and this makes it difficult to compare study findings and formulate reliable guidance.

The most robust findings show that female ultramarathon runners have superior fatigue resistance, greater susceptibility to bone stress injuries and the athlete triad, and disparate oxidative responses compared with males. When considered as a whole, the body of research currently suggests that sex-specific recommendations and guidelines could improve performance and health outcomes in female ultramarathon runners. However, the evidence base is currently insufficient to formulate such guidelines, and further research that recognises sex as an important bivariate measure is required. Specifically, there is a dearth of interventional studies examining sex differences in the physiological responses to different training, nutrition, and recovery modalities. Study designs that investigate the effects of interventions during different stages of the menstrual cycle are particularly needed. Future research should also compare males with both pre- and post-menopausal females, investigate the effects of female sex hormones on fluid balance and EAH risk, and clarify the long-term effects of AO supplementation on female ultramarathon performance. It is hoped that increasing female participation in ultramarathons will be reflected by larger female cohorts in future studies, allowing for more direct comparisons between male and female physiology, and the development of scientifically robust recommendations for female ultramarathon athletes.

## Data Availability

Data sharing is not applicable to this article as no datasets were generated or analysed during the current study.

## Abbreviations

AKI: Acute kidney injury
AO: Antioxidant
AST: Aspartate transaminase
BF%: Body fat percentage
BMD: Bone mineral density
BMI: Body mass index
BP: Blood pressure
BSI: Bone stress injury
CG: Carbonyl groups
CK: Creatine kinase
CPET: Cardiopulmonary exercise test
CPK: Creatine phosphokinase
CRP: C-reactive protein
CV: Coefficient of variability
DEXA: Dual-energy X-ray absorptiometry
DNA: Deoxyribonucleic acid
EAH: Exercise-associated hyponatraemia
EAMC: Exercise-associated muscle cramps
ECHO: Echocardiography
EEG: Electroencephalography
EF: Ejection fraction
EICF: Exercise induced cardiac fatigue
F2IsoP: F2 isoprostanes
FEV1: Forced expiratory volume over 1 s
FFA: Free fatty acids
FVC: Forced vital capacity
GLS: Global longitudinal strain
GPx: Glutathione peroxidase
GR: Glutathione reductase
Hct: Haematocrit
HR: Heart rate
HRI: Heat-related illness
HRV: Heart rate variability
IL-6: Interleukin 6
IMVC: Isometric maximal voluntary contraction
LA: Left atrium
LDH: Lactate dehydrogenase
LV: Left ventricle
MDA: Malondialdehyde
MFO: Maximal fat oxidation rate
MIP: Mean inspiratory pressure
%MRS: Percentage of mean running speed
MSK: Musculoskeletal
MVC: Maximal voluntary contraction
PB: Personal best
PEF: Peak expiratory flow
RA: Right atrium
RCT: Randomised controlled trial
ROS: Reactive oxygen species
RV: Right ventricle
RVFWS: Right ventricular free wall strain
sIgA: Salivary immunoglobulin A secretion
SSR: Significant speed reduction
TNF-α: Tumour necrosis factor alpha
URTI: Upper respiratory tract infection
UTMB: Ultra-Trail du Mount-Blanc
V̇O_2_max: Maximal oxygen uptake
Vpeak: Peak speed reached during cardiopulmonary exercise testing
VT1: First ventilatory threshold
VT2: Second ventilatory threshold
